# Actinomycetes: A Repertory of Green Catalysts with a Potential Revenue Resource

**DOI:** 10.1155/2013/264020

**Published:** 2013-04-18

**Authors:** Divya Prakash, Neelu Nawani, Mansi Prakash, Manish Bodas, Abul Mandal, Madhukar Khetmalas, Balasaheb Kapadnis

**Affiliations:** ^1^Dr. D. Y Patil Biotechnology & Bioinformatics Institute, Dr. D. Y. Patil Vidyapeeth, Pune 411 033, India; ^2^System Biology Research Center, School of Life Sciences, University of Skövde, P.O. Box 408, 541 28 Skövde, Sweden; ^3^Department of Microbiology, University of Pune, Pune 411 007, India

## Abstract

Biocatalysis, one of the oldest technologies, is becoming a favorable alternative to chemical processes and a vital part of green technology. It is an important revenue generating industry due to a global market projected at $7 billion in 2013 with a growth of 6.7% for enzymes alone. Some microbes are important sources of enzymes and are preferred over sources of plant and animal origin. As a result, more than 50% of the industrial enzymes are obtained from bacteria. The constant search for novel enzymes with robust characteristics has led to improvisations in the industrial processes, which is the key for profit growth. Actinomycetes constitute a significant component of the microbial population in most soils and can produce extracellular enzymes which can decompose various materials. Their enzymes are more attractive than enzymes from other sources because of their high stability and unusual substrate specificity. Actinomycetes found in extreme habitats produce novel enzymes with huge commercial potential. This review attempts to highlight the global importance of enzymes and extends to signify actinomycetes as promising harbingers of green technology.

## 1. Introduction

Biocatalysis offers green and clean solutions to chemical processes and is emerging as a challenging and reverred alternative to chemical technology. The chemical processes are now carried out biologically by biocatalysts (enzymes) which are integral components of any biological system. However, the utility of enzymes is not naïve to us, as they have been an integral part of our lives from immemorial times. Their utility dates back to 1914, when they were used in detergents even before their protein nature was determined in 1960 [1]. Their use in fermentation processes like wine and beer manufacture, vinegar production, and bread making has been practised for several decades. However, a commercial breakthrough happened in the later half of 20th century with first commercial protease production in 1957 by Novozymes [1]. Since then, due to the advent of newer industries, the enzyme industry has not only seen an enormous growth but has also matured with a technology-oriented perspective.

Commercially available enzymes are derived from plants, animals, and microorganisms. The enzymes derived from plants include papain, bromelain, ficin, lipooxygenase, among several others [2], and those derived from animal sources include pepsin and renin. However, a major fraction of commercially available enzymes are derived from microbes due to their ease of growth, nutritional requirements, and downstream processing. To meet the increasing demand of robust, high turnover, economical and easily available biocatalysts, research is always channelized for novelity in enzyme or its source or for improvement of existing enzymes by engineering at gene and protein level [1]. Search for novel enzymes from unusual ecological niches is often more attractive option leading to development of high-throughput screening programs. Enzymes with new physical and physiological characteristics like high productivity, specificity, stability at extreme temperature, pH or other physiological conditions, low cost of production, and tolerance to inhbitors are always most sought after properties from an industrial standpoint. An important criterion for enzymes derived from microbes remains that the source microbe should have a generally regarded as safe (GRAS) status [3].

 Many microbes particularly bacteria and fungi are currently employed for the production of various industrial enzymes [4]. Hydrolases cover more than 75% of commercially used enzymes and are often in great demand. These are however used in a crude form to make the process economically viable and also to meet the demand of enzyme at a large scale [5]. Amongst the hydrolases, proteases occupy an important platform, as they are extensively used in detergent industry, followed by starch industry which is the second largest user of enzymes and textiles, baking, food, and animal feed industries. The applications of few commercially significant enzymes are enlisted in Table 1.

This review touches the global and Indian enzyme market scenario and further highlights the potential of actinomycetes as sources of important industrial enzymes like cellulases, pectinases, proteases, and chitinases which can be employed for the recovery of numerous value added products with applications in biomedicine and waste management.

## 2. Enzyme Market: Global and Indian Scenario

The global market is expected to experience a growth of 6% in enzyme requirement with an estimated market of $7 billion in 2013 [28]. North America and Western Europe are predicted to show an increased growth, while the highest growth is likely in developing countries of Asian, African and Mideast regions, along with Latin America and Eastern Europe. China is emerging as an important base and market for industrial enzymes due to various R&D activites set up by many industrial giants which accounts for 10% of the global scenario [29]. The demand of diagnostic and therapeutic enzymes is expected to increase owing to improvement in medical care facilities in developing countries and global health care reforms. Few enzyme manufacturing industries in the world include AB Enzymes GmbH, Advanced Enzyme Technologies Ltd., Amano Enzyme Inc., Asahi Kasei Pharma Corporation, Cargill Texturizing Solutions, Genencor International Inc., DSM Food Specialties, Hayashibara Company, Nexgen Biotechnologies Inc., Novozymes A/S, and Maps Enzymes Ltd.

**Figure 1:**
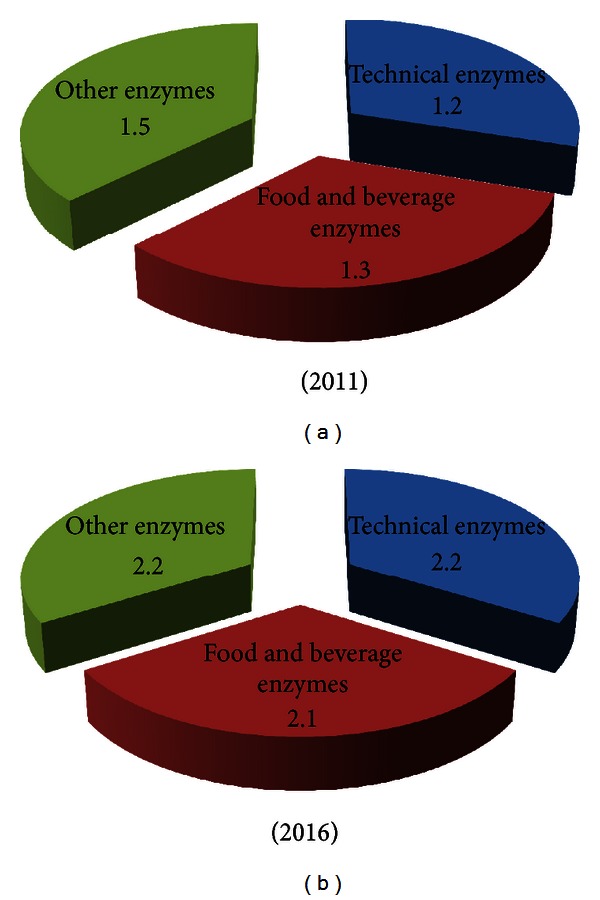
Global enzyme industry market in the years 2011 and 2016.

**Figure 2:**
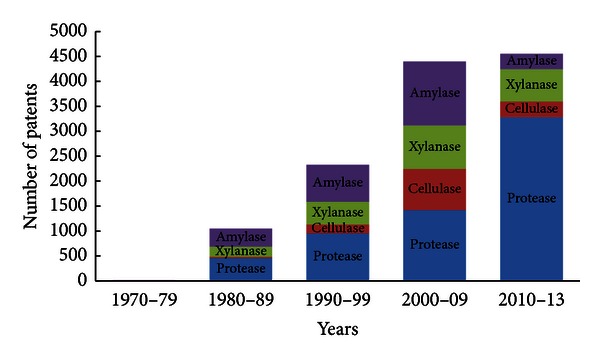
Growth in number of patents issued for important industrial enzymes over past few decades.

The global industrial enzyme market has evolved continounsly due to numerous mergers and acquisitions. In the year 2011, enzyme industry giants like Novozymes and DuPont occupied market shares of 47% and 21%, respectively [30]. Technical enzymes were valued at $1.2 billion in 2011, and this is expected to rise to $2.2 billion in 2016 with the highest sales predicted in the leather and bioethanol markets [31]. Similarly, food and beverage enzyme sector is expected to achieve about $2.1 billion by 2016, from a value of $1.3 billion in 2011 as shown in Figure 1. This is well correlated with the numerous patents which have been filed over a period of years which indicate an increasing trend. From Figure 2, it can be inferred that due to the lack of information on intellectual property rights (IPRs) in the 1970s, there were hardly any patents on any of the industrial enzymes [32]. But from the year 2000 till date, there has been a tremendous increase in the number of patents filed or obtained for various enzymes. The maximum number of patents is for proteases followed by amylases and cellulases perhaps due to maximum utility of these enzymes. The application and issuing of patents for various enzymes is expected to grow in the future due to green technologies.

Speciality enzymes due to their unique properties like extreme thermostability, specific activity, and activity over a wide range of pH are expected to occupy an important category in future due to their robustness. These speciality enzymes also include those useful in medicine and biotechnology, for example, kinases, polymerases, and nucleases [33]. Besides this, their utility in wide range of personal care products is revolutionalizing the cosmetic industry too. The speciality sector is expected to reach $4.3 billion by 2015, and industrial enzyme segment is worth $80 million, according to reports by reputed market researchers and industry analysts [34]. This growth would be mainly accelerated by the pharmaceutical and diagnostics industry, the largest end-users of these enzymes which will continue to grow due to the emergence of enzyme replacement therapies and innovations in thrombolytics.

India imports 70% of the total enzyme consumed by its market which indicates need of indigenous manufacturers and technologies. The most important enzymes in demand are of the pharmaceutical sector consuming more than 50% of the total enzymes. This is followed by detergent enzymes (20%), textile enzymes (20%), and the rest comprises of food enzymes [35]. Novozymes is one of the leading industries in the enzyme market in India. The need of the hour is a strong R&D in terms of investment. There is necessity for strong legislation and IPR regulations which would make India withstand the stiff competition on the global front. In addition, the enzyme market is risky and has high captial costs. India offers excellent human resource power which can lower production costs compared with many other countries making this country an attractive location for investment. Besides, the biodiversity in India is valuable for the screening of novel enzymes and metabolites which can be produced and utilized at the industrial level.

## 3. Actinomycetes as a Source for Industrial Enzymes

Actinomycetes are one of the ubiquitous dominant groups of gram positive bacteria. Actinomycetes have been commercially exploited for the production of pharmaceuticals, neutraceuticals, enzymes, antitumor agents, enzyme inhibitors, and so forth [36]. These bioactive compounds are of high commercial value, and hence actinomycetes are regularly screened for the production of novel bioactive compounds. A wide array of enzymes and their products applied in biotechnological industries and biomedical fields has been reported from various genera of actinomycetes. Since there is vital information available due to the advent of genome and protein sequencing data, actinomycetes have been continuously employed of the production of proteases, cellulases, chitinases, amylases, xylanases, and others. Representative examples of industrially important enzymes from actinomycetes are discussed below, and their enzymatic properties are enlisted in Table 2.

### 3.1. Cellulases

Cellulases convert cellulose to fermentable sugars fit for human consumption and the largest known producers are from genus *Streptomyces* [37]. Cellulases from *Streptomyces* sp. are reported to have an alkaline pH optimum and high thermostability. Subsequently, the enzyme was used as a supplement in detergents to clean, soften, and restore the color of the fabrics. It was also tested for the treatment of textiles, processing of paper and pulp, and as an animal feed additive [6]. Besides *Streptomyces*, several other genera like *Thermobifida* and *Micromonospora* produce recombinant cellulases that can be commercially exploited [7]. A recombinant cellulase with thermal and pH stability is reported from *Streptomyces thermoviolaceus*; this enzyme retains its activity in the presence of commercial detergents highlighting its superiority to the existing commercial cellulases [8]. Cellulase from *Thermomonospora fusca* has been used for degradation of cotton and avicel [9]. These enzymes not only hold a biotechnological promise but can be economical due to their low cost of production. Their production can be carried out on cheap substrates like rice and wheat straw [10] and fruit peels [38].

### 3.2. Xylanases

*Streptomyces* spp. are prolific producers of another commercially important enzyme, xylanase. Xylanases from *Streptomyces* sp. are preferred in treatment of rice straw pulp to improve the pulp bleachability. This preference is due to absence of cellulase contamination in the xylanase and also due to reduced usage of chemicals during bleaching and pulping [39]. Further, xylanases from actinomycetes are stable on kraft pulps and can be used in the crude form thereby making the process economical. High thermostability and specific activity, two desirable properties of enzymes to be employed in industrial processes, are reported in xylanases from *Actinomadura* sp. FC7 and *Nonomuraea flexuosa* [11, 12]. Similarly, fused xylanases from fungi and actinomycetes have been employed in paper and pulp industries, due to high thermal and pH stability [13]. In many higher plants and agricultural wastes, the content of xylan is almost 20–40% of the dry weight. Xylan with hemicelluloses is the second most renewable biopolymer [14]. Since pure xylan is expensive, alternate cheap substrates like these can serve as potential substrates at industrial level. *Streptomyces* spp. were able to produce high levels of xylanase when untreated rice straw was utilized which resulted in significant biobleaching [39]. Similarly, *Streptomyces* sp. was able to hydrolyze various agricultural residues like oil cake and straw waste which resulted in increased biogas production [14].

### 3.3. Amylases

Another important group of enzymes is the amylases which are employed in the starch processing industry for the conversion of starch to high fructose syrups [15]. One of the focus areas with respect to starch industry is the production of tailor length maltooligosaccharides which can be produced with amylases with a very specific mode of action. Thermophilic and acidophilic amylases which can find applications in bakery, brewing, and alcohol industries have been studied from *Streptomyces erumpens* [16]. Thermostable amylases are reported from *Nocardiopsis* sp. which have important applications in bakery and paper industries [17]. The amylase from *Thermobifida* sp. produced maltotriose as the major end product from refined starch and raw sago starch [18]. Such amylases are lucrative catalysts in nutrition and healthcare [18]. Besides this, end-product specific amylases can be used for the production of maltooligosaccharides from low cost starch substrates [40]. Many actinomycetes have also been reported for the production of cold-active *α*-amylases which can be employed in textile industries, detergents, bioethanol producing industries [19]. 

### 3.4. Pectinases

Food industry uses pectinases particularly in clarification of fruit juices, in degumming of fibres, wine making, and retting of bast fibres. Pectinases from *Streptomyces* sp. are reported [20]; however, reports of pectinases from other genera of actinomycetes are scanty. The demand of cold-active pectate lyases is increasing due to their ability to retain the palatability and nutritional characteristics of food products. Occurrence of pectin degrading genes in few actinomycetes suggests their characterization could perhaps yield pectinases with novel properties. 

### 3.5. Proteases

The quest for novel proteases and their formulations for industries like detergents, animal feed, and breweries is observed from several decades. Most of the proteases reported from *Streptomyces* spp. are alkali-tolerant, and some of them are salt tolerant and belong to genera other than the genus *Streptomyces* [21]. Proteases from *Nocardiopsis* spp. are employed as detergents additives [22] and for the depilation of hides and skins in the leather industry. Dehairing of goat skin by proteases from *Streptomyces* sp. makes the process economically and environmentally feasible [23]. Keratin-rich wastes like feathers, hair, nails, and horn are waste products of agroindustrial processes. Keratinolytic *Streptomyces* spp. capable of degrading keratin at temperatures higher than 50°C are reported [24]. Some *Streptomyces* hydrolyze keratin by pronases as seen for *Streptomyces griseus* [24]. Proteases from other sources are used in conjunction with enzymes from actinomycetes for recovery of antioxidants from shellfish waste. Protease production was also carried by growing *Microbispora* sp. on the shellfish waste [25]. End products of protein hydrolysis rich in amino acids and peptides serve as a low cost animal feed. 

### 3.6. Chitinases

Chitinases are another class of hydrolases which have gained tremendous importance in the past two decades. They are glycosyl hydrolases that catalyze the degradation of chitin, which is an insoluble linear **β**-1,4-linked polymer of *N*-acetylglucosamine (GlcNAc). Chitin is a major constituent of the shells of crustaceans, exoskeletons of insects, and cell walls of a variety of fungi [26]. Chitinases are useful in protoplast preparation from fungi, as biocontrol agents against plant pathogenic fungi, nematodes, and so forth and are recently used for the extraction of chitin oligomers which are important biomedical products. Chitinases occur in several actinomycetes and possess unique properties in terms of thermostability and activity in wide pH range which makes them suitable for industrial applications [21, 26, 27]. One of their most resourceful applications is the production of chitin oligosaccharides. Chitin oligosaccharides (COS) have anticoagulant, antimicrobial, anticholesteremic, anticancer, wound-healing, antitumor, and antioxidant activities which make them bright candidates for biomedical applications [26]. COS can be recovered from low cost substrates like shrimp, crab, and squid pen waste [41, 42]. Chitinase from *Microbispora* sp. was employed for the recovery of chitobiose, a potential antioxidant which can be used as a food additive and for other biomedical applications [25]. This renewable resource can be utilized for the growth of many chitinolytic organisms as well as for the effective recovery of COS at the industrial level. The disposal of the waste is also carried out effectively by the biological utilization by actinomycetes.

### 3.7. Other Enzymes from Actinomycetes

An array of other enzymes with industrial potential reported from actinomycetes includes lignin peroxidases, laccases, and tyrosinases which are effective in the treatment of textile dyes [43**]** promising their application in waste treatment plants. Esterases and amidases from *Nocardia* sp. have been used to increase the hydrophilicity of polyethylene terephthalate and polyamide fibers. This can be an ecofriendly and cost-effective method in textile industries [43]. To get enzymes with novel properties or functionalities, high throughput screening (HTS) programs are adopted for choosing rare actinomycetes which are a source of novel compounds. Success examples include therapeutic enzymes like thrombinase and L-asparaginase from marine *Streptomyces* sp. which are used in the treatment of myocardial infarction and leukemia. A sponge associated *Streptomyces* sp. produced phytoene, a carotenoid with enhanced antioxidant activity making it a promising food additive [44]. 

Access to advanced technologies has made it possible to obtain untapped microbes with novel properties, and actinomycetes have been natural reservoirs of excellent enzymes. The existing HTS methods are used for choosing industrially important bacteria and have not been actively extended to actinomycetes. Several HTS methods which can be used for exploring novel enzymes from actinomycetes are discussed. Fluorescence activated cell sorting (FACS) is successfully employed for sorting of desired clones from a genomic library, where fluorescent substrate specific for a particular enzyme is used. The positive fluorescence indicates biocatalytic activity of the clone [45]. Gel MicroDrop technology detects clones positive for specific enzymes by capturing the fluorescence emitted due to catalytic breakdown of biotinylated substrate by the clone [46]. 

Metagenomics has offered rapid screening methods where the bioactive potential of unculturable microbes can be explored. A clonal library is prepared using the metagenome obtained from extreme habitats like arid regions, ocean beds, stratosphere, and others without expecting the actual microbes to grow under laboratory conditions which usually limits the exploitation of the bioactive potential of these nonculturable microbes [47]. These technologies also help to determine the functional aspects of a microenvironment [48]. Although metagenomic approach has many advantages, it suffers from a common disadvantage like low or no expression of desired gene(s). Multiple displacement amplification has allowed researchers to overcome the problem of low or no expression. Here whole genome amplification is carried out from single cells, which unlocks entire biochemical potential of an uncultured microbe from a complex habitat. Other innovative approaches like substrate-induced gene expression screening (SIGEX), preamplification inverse-PCR (PAI-PCR), and metagenomic DNA shuffling provide insights on the functional metagenomics of a particular habitat [48].

 A smart and diversified technique which accelerates evolution is the “directed evolution” approach, where a library of genetic variants is created and the mutants are screened for desired enzymatic properties. The best variants are shortlisted and reorganized for another round of library creation which is repeated a number of times to possibly get the best variants, a process which follows the rule of natural evolution but is performed at a pace that gives the result of evolution in a short time [49]. This method is a significant driving force for the discovery of next generation biocatalysts [50]. An added advantage to this method is the inclusion of ultrahigh-throughput FACS-based screening which aids in the rapid screening of the variant library [51]. Recent improvements in screening technologies which can be very useful for enzymes from actinomycetes are the Drop-based microfluidics which was successfully used to screen mutants with ten times potent horse peroxidase activity than wild type [52]. HTS in drop-based microfluid platform carries a small foot print chip with an array of insoluble substrates specific for the enzyme of interest; this gives rapidity, parallel execution, and cost economics to the screening protocol [53]. 

Reporter gene technology is another step up in the screening methods which offer simplicity and sensitivity. Green fluorescent protein (GFP), a reporter of choice, is widely used due to ease of its detection and acquiescence with host systems [54], and coupled hexose oxidases are used for the detection of reducing sugars released from polymeric substrates due to enzyme hydrolysis [55]. A workhorse in proteomics, MALDI-LTQ-Orbitrap, is an excellent tool to screen proteins in complex matrices and suspensions. The technique works on ion trap and MALDI and is interfaced with liquid and gas chromatography separations [56]. Other ionization techniques include electrospray ionization mass spectrometry (ESI-MS) which is used to measure femtomole quantities of proteins [57]. Some *in silico* techniques to sort potent enzymes from database are developed. Predictive 3D-QSAR CoMFA and CoMSIA are powerful techniques which predict superior enzymes based on structural properties and microenvironment provided for *in silico* reaction with substrate. These techniques however require validation in laboratory to assess the suitability of the models in prediction [58]. The extension of these advanced technologies in screening of bioactive molecules from actinomycetes might provide supreme strains and enzymes with premium properties.

## 4. Conclusion

The industrial enzyme market is one of the fastest growing revenue generating sectors in the world. Only 20 enzymes are currently utilized on the industrial level indicating the need for further research and development of low cost enzymes and their production. The application of enzymes in diverse biotechnological industries indicates a positive trend which needs to be satisfied with the discovery of novel enzymes and metabolites. Since very few enzymes have been potentially utilized at the industrial level; there is a huge scope for the development of robust and low cost enzymes. Actinomycetes are a reservoir of important enzymes and metabolites due to their versatile genetic repertory. However, many of the rare genera of actinomycetes have been neither explored nor manipulated for their biotechnological and industrial potential. Studies on unique ecological environments could yield molecules that could become future harbingers of green technology.

## Figures and Tables

**Table 1:** Commercially relevant enzymes produced by actinomycetes.

Enzyme	Use	Industry of application
Protease	Detergents	Detergent
	Cheese making	Food
	Clarification- low calorie beer	Brewing
	Dehiding	Leather
	Treatment of blood clot	Medicine

Cellulase	Removal of stains	Detergent
	Denim finishing, softening of cotton	Textile
	Deinking, modification of fibers	Paper and pulp

Lipase	Removal of stains	Detergent
	Stability of dough and conditioning	Baking
	Cheese flavoring	Dairy
	Deinking, cleaning	Textile

Xylanase	Conditioning of dough	Baking
	Digestibility	Animal feed
	Bleach boosting	Paper and pulp

Pectinase	Clarification, mashing	Beverage
	Scouring	Textile

Amylase	Removal of stains	Detergent
	Softness of bread softness and volume	Baking
	Deinking, drainage improvement	Paper and pulp
	Production of glucose and fructose syrups	Starch industry
	Removal of starch from woven fabrics	Textile

Glucose oxidase	Strengthening of dough	Baking

Lipoxygenase	Bread whitening	Baking

Phytase	Phytate digestibility	Animal feed

Peroxidase	Removal of excess dye	Textile

**Table 2:** List of industrially viable enzymes from actinomycetes and their characteristics.

Enzyme	Producing Strain	pH stability	Thermal stability	Substrate specificity	Reference
	Recombinant *Streptomyces* sp.	5.0–12.0	40–50°C	CMC	[6]
	*Thermobifida * *halotolerans *	6.0–8.0	40–50°C	CMC	[7]
Cellulase	Recombinant *Streptomyces* sp.	10.0	40°C	CMC	[8]
	*Thermomonospora* sp.	7.0–10.0	50°C	CMC	[9]
	*Streptomyces * *ruber *	5.5–7.0	35–40°C	CMC	[10]

	*Actinomadura* sp.	4.0	70°C	Xylan	[11]
Xylanase	Recombinant strain	5.0–7.0	70–80°C	Xylan	[12]
	Recombinant strain	5.0–7.0	60–70°C	Birch xylan	[13]
	*Streptomyces* spp.	8.0–11.0	45–60°C	Xylan	[14]

	*Streptomyces *sp.	5.0–7.0	45–50°C	Starch	[15]
	*Streptomyces * *erumpens *	9.0-10.0	40–50°C	Starch	[16]
Amylase	*Nocardiopsis* sp.	8.6	70–80°C	Starch	[17]
	*Thermobifida * *fusca *	5.0–7.0	60°C	Starch	[18]
	*Nocardiopsis* sp.	5.0–10.0	35–45°C	Starch	[19]

Pectinase	*Streptomyces * *lydicus *	4.0–7.0	45°C	Polygalacturonic acid	[20]

	*Thermoactinomyces* sp.	4.0	50°C	NA	[21]
	*Nocardiopsis* sp.	10.0	40–50°C	Casein	[22]
Protease	*Streptomyces * *pactum *	7.5	40°C	Casein	[23]
	*Streptomyces * *thermoviolaceus *	6.5	65°C	Keratin	[24]
	*Streptomyces *sp*. *	4.0–11.0	30–60°C	Keratin azure	[25]

	*Nocardiopsis * *prasina *	7.0	50–60°C	Colloidal chitin	[21]
Chitinase	*Streptomyces thermoviolaceus *	6.0	60°C	Colloidal chitin	[26]
	*Microbispora * sp.	3.0–11.0	30–50°C	Colloidal chitin	[27]

^CMC: Carboxymethyl cellulose; NA: not available.^

## References

[1] Leisola M, Jokela J, Pastinen O, Turunen O, Schoemaker HE (2004). Industrial use of enzymes. *In Encyclopedia of Life Support Systems (EOLSS)*.

[2] http://naturebiochem.com/downloads/Enzymes_Pharma_applications.

[3] http://ec.europa.eu/food/fs/sc/scan/out85_en.pdf.

[4] Monteiro de souza P, de Oliveira P (2010). Application of microbial a-amylase in industry: a review. *Brazilian Journal of Microbiology*.

[5] http://ec.europa.eu/environment/archives/dansub/pdfs/enzymerepcomplete.pdf.

[6] Jones BE, Kleij Wilhelmus AH, Van Solingen P, Weyler W Cellulase producing actinomycetes, cellulase produced there from and method of producing same.

[7] Zhang F, Chen JJ, Ren WZ (2011). Cloning, expression and characterization of an alkaline thermostable GH9 endoglucanase from *Thermobifida halotolerans* YIM, 90462 T. *Bioresource Technology*.

[8] Jones BE, Kleij Wilhelmus AH, Van Solingen P, Weyler W Cellulase producing actinomycetes, cellulase produced there from and method of producing same.

[9] George SP, Ahmad A, Rao MB (2001). Studies on carboxymethyl cellulase produced by an alkalothermophilic actinomycete. *Bioresource Technology*.

[10] El-Sersy NA, Abd-Elnaby H, Abou-Elela GM, Ibrahim HAH, El-Toukhy NMK (2010). Optimization, economization and characterization of cellulase produced by marine *Streptomyces ruber*. *African Journal of Biotechnology*.

[11] Brzezinski R, Dery CV, Beaulieu C Thermostable xylanase DNA, protein and methods in use.

[12] Zhang J, Siika-Aho M, Puranen T, Tang M, Tenkanen M, Viikari L (2011). Thermostable recombinant xylanases from *Nonomuraea flexuosa* and *Thermoascus aurantiacus* show distinct properties in the hydrolysis of xylans and pretreated wheat straw. *Biotechnology for Biofuels*.

[13] Fagerstrom R, Lahtinen T, Lantto R Production and secretion of actinomycete xylanases in a filamentous trichoderma fungus.

[14] Priya BS, Stalin T, Selvam K (2012). Efficient utilization of xylanase and lipase producing thermophilic marine actinomycetes (*Streptomyces albus* and *Streptomyces hygroscopicus*) in the production of ecofriendly alternative energy from waste. *African Journal of Biotechnology*.

[15] Ammar YB, Matsubara T, Ito K (2002). New action pattern of a maltose-forming *α*-amylase from *Streptomyces* sp. and its possible application in bakery. *Journal of Biochemistry and Molecular Biology*.

[16] Kar S, Ray RC (2008). Statistical optimization of *α*-amylase production by Streptomyces erumpens MTCC 7317 cells in calcium alginate beads using response surface methodology. *Polish Journal of Microbiology*.

[17] Stamford TLM, Stamford NP, Coelho LCBB, Araújo JM (2001). Production and characterization of a thermostable *α*-amylase from *Nocardiopsis* sp. endophyte of yam bean. *Bioresource Technology*.

[18] Yang C-H, Liu W-H (2004). Purification and properties of a maltotriose-producing alpha-amylase from *Thermobifida fusca*. *Enzyme and Microbial Technology*.

[19] Kuddus M, Roohi JM, Arif JM, Ramteke PW (2011). An overview of cold-active microbial alpha-amylase: adaptation strategies and biotechnological potentials. *Biotechnology*.

[20] Jacob N, Asha Poorna C, Prema P (2008). Purification and partial characterization of polygalacturonase from *Streptomyces lydicus*. *Bioresource Technology*.

[21] Horikoshi K (1999). Alkaliphiles: some applications of their products for biotechnology. *Microbiology and Molecular Biology Reviews*.

[22] Moreira KA, Albuquerque BF, Teixeira MFS, Porto ALF, Lima Filho JL (2002). Application of protease from *Nocardiopsis* sp. as a laundry detergent additive. *World Journal of Microbiology and Biotechnology*.

[23] Mitra P, Chakrabartty PK (2005). An extracellular protease with depilation activity from *Streptomyces nogalator*. *Journal of Scientific and Industrial Research*.

[24] Brandelli A (2008). Bacterial keratinases: useful enzymes for bioprocessing agroindustrial wastes and beyond. *Food and Bioprocess Technology*.

[25] Jaouadi B, Abdelmalek B, Fodil D (2010). Purification and characterization of a thermostable keratinolytic serine alkaline proteinase from *Streptomyces* sp. strain AB1 with high stability in organic solvents. *Bioresource Technology*.

[26] Bhattacharya D, Nagpure A, Gupta RK (2007). Bacterial chitinases: properties and potential. *Critical Reviews in Biotechnology*.

[27] Nawani NN, Kapadnis BP, Das AD, Rao AS, Mahajan SK (2002). Purification and characterization of a thermophilic and acidophilic chitinase from *Microbispora* sp. V2. *Journal of Applied Microbiology*.

[28] http://www.reportlinker.com/p0747897-summary/World-Enzymes-Industry.html.

[29] The Freedonia group (2011). Industry Study with Forecasts for 2015 and 2020. *Study*.

[30] Dewan SS Global markets for enzymes in industrial applications (BIO030G).

[31] Dewan SS Global Markets and Technologies for Biofuel Enzymes (EGY099A).

[32] http://www.wipo.int.

[33] http://www.specialtyenzymes.com/seb-group-usa.

[34] http://www.newstatesman.com/healthcare-and-pharmaceuticals/2010/11/specialty-enzymes-global.

[35] http://forbesindia.com/article/breakpoint/novozyme-makes-enzymes-for-a-better-lifes/32760/1.

[36] Remya M, Vijayakumar R (2008). Isolation and characterization of marine antagonistic actinomycetes from west coast of India. *Medicine and Biology*.

[37] Jang HD, Chang KS (2005). Thermostable cellulases from *Streptomyces* sp.: scale-up production in a 50-l fermenter. *Biotechnology Letters*.

[38] Rathan RK, Ambili M (2011). Cellulase Enzyme Production by Streptomyces Sp. Using Fruit Waste as Substrate. *Australian Journal of Basic and Applied Sciences*.

[39] Rifaat HM, Nagieb ZA, Ahmed YM (2006). Production of xylanases by *Streptomyces* species and their bleaching effect on rice straw pulp. *Applied Ecology and Environmental Research*.

[40] Kikani BA, Singh SP (2011). Single step purification and characterization of a thermostable and calcium independent *α*-amylase from *Bacillus amyloliquifaciens* TSWK1-1 isolated from Tulsi Shyam hot spring reservoir, Gujarat (India). *International Journal of Biological Macromolecules*.

[41] Jeon YJ, Park PJ, Kim SK (2001). Antimicrobial effect of chitooligosaccharides produced by bioreactor. *Carbohydrate Polymers*.

[42] Wang SL, Hsu WH, Liang TW (2010). Conversion of squid pen by Pseudomonas aeruginosa K187 fermentation for the production of N-acetyl chitooligosaccharides and biofertilizers. *Carbohydrate Research*.

[43] Heumann S, Eberl A, Pobeheim H (2006). New model substrates for enzymes hydrolysing polyethyleneterephthalate and polyamide fibres. *Journal of Biochemical and Biophysical Methods*.

[44] Jayaprakashvel M (2012). Therapeutically active biomolecules from marine actinomycetes. *Journal of Modern Biotechnology*.

[45] Short JM, Keller M High throughput screening for novel enzymes.

[46] Keller M, Lafferty MW, Short MJ High throughput or capillary-based screening for a bioactivity or biomolecules.

[47] Lorenz P, Liebeton K, Niehaus F, Eck J (2002). Screening for novel enzymes for biocatalytic processes: accessing the metagenome as a resource of novel functional sequence space. *Current Opinion in Biotechnology*.

[48] Kennedy J, Flemer B, Jackson SA (2010). Marine metagenomics: new tools for the study and exploitation of marine microbial metabolism. *Marine Drugs*.

[49] Olsen M, Iverson B, Georgiou G (2000). High-throughput screening of enzyme libraries. *Current Opinion in Biotechnology*.

[50] Nannemann DP, Birmingham WR, Scism RA, Bachmann BO (2011). Assessing directed evolution methods for the generation of biosynthetic enzymes with potential in drug biosynthesis. *Future Medicinal Chemistry*.

[51] Yang G, Withers SG (2009). Ultrahigh-throughput FACS-based screening for directed enzyme evolution. *ChemBioChem*.

[52] Agrestia JJ, Antipov E, Abatea AR (2010). Ultrahigh-throughput screening in drop-based microfluidics for directed evolution. *Proceedings of the National Academy of Sciences of the USA*.

[53] Chang C, Sustarich J, Bharadwaj R, Chandrasekaran A, Adams PD, Singh AK (2013). Droplet-based microfluidic platform for heterogeneous enzymatic assays. *Lab Chip*.

[54] Paley O, Agnello G, Cantor J, Yoo TH, Georgiou E Stone G (2013). GFP reporter screens for the engineering of amino acid degrading enzymes from libraries expressed in bacteria. *Methods in Molecular Biology*.

[55] Ostafe R, Prodanovic R, Commandeur U, Fischer R (2013). Flow cytometry-based ultra-high-throughput screening assay for cellulase activity. *Analytical Biochemsitry*.

[56] Akeroyd M, Olsthoorn M, Gerritsma J (2013). Searching for microbiall protein over-expression in a complex matrix using automated high throughput MS-based proteomics tools. *Journal of Biotechnology*.

[57] Smith C, Li X, Mize T (2013). Sensitive, high throughput detection of proteins in individual, surfactant stabilized picoliter droplets using NanoESI mass spectrometry. *Analytical Chemistry*.

[58] Murumkar PR, Gupta SD, Zambre VP, Giridhar R, Yadav MR (2009). Development of predictive 3D-QSAR CoMFA and CoMSIA models for *β*-aminohydroxamic acid-derived tumor necrosis factor-*α* converting enzyme inhibitors. *Chemical Biology and Drug Design*.

